# Treatment Options for COVID-19: A Review

**DOI:** 10.3389/fmed.2020.00480

**Published:** 2020-07-31

**Authors:** Mukarram Jamat Ali, Muhammad Hanif, Muhammad Adnan Haider, Muhammad Umer Ahmed, FNU Sundas, Arham Hirani, Izhan Ali Khan, Khurram Anis, Amin H. Karim

**Affiliations:** ^1^Department of Medicine, King Edward Medical University, Lahore, Pakistan; ^2^Department of Medicine, Khyber Medical College, Peshawar, Pakistan; ^3^Department of Medicine, Allama Iqbal Medical College, Lahore, Pakistan; ^4^Department of Medicine, Ziauddin University, Karachi, Pakistan; ^5^Department of Medicine, Dow University of Health Sciences, Karachi, Pakistan; ^6^Department of Gastroenterology, Pakistan Kidney and Liver Institute, Lahore, Pakistan; ^7^Department of Cardiology, Baylor College of Medicine, Houston, TX, United States

**Keywords:** COVID-19, SARS-CoV-2, chloroquine/hydroxychloroquine, ivermectin, remdesivir, immunoglobulin, tocilizumab

## Abstract

**Background:** The recent COVID-19 pandemic sweeping the globe has caused great concern worldwide. Due to the limited evidence available on the dynamics of the virus and effective treatment options available, severe acute respiratory syndrome coronavirus 2 (SARS-CoV-2) has had a huge impact in terms of morbidity and mortality. The economic impact is still to be assessed.

**Aims:** The purpose of this article is to review the evidence for the multiple treatment options available, to consider the future of this global pandemic, and to identify some potential options that could revolutionize the treatment of COVID-19. Moreover, this article underscores the sheer importance of repurposing some of the available antiviral and antimicrobial agents that have long been in use so as to have an effective and expeditious response to this widespread pandemic and the need to conduct a multicenter global randomized controlled trial to find an effective single antiviral agent or a cocktail of available antimicrobial agents.

**Method:** We thoroughly searched and reviewed various case reports, retrospective analyses, and *in vitro* studies published in PubMed, EMBASE, and Google Scholar regarding the treatment options used for SARS-CoV, MERS-CoV, and SARS-CoV-2 since its outbreak in an attempt to highlight treatments with the most promising results.

**Conclusion:** We are currently facing one of the worst pandemics in history. Although SARS-CoV-2 is associated with a lower mortality rate than are SARS-CoV and MERS-CoV, its higher infectivity is making it a far more serious threat. Unfortunately, no vaccine against SARS-CoV-2 or effective drug regimen for COVID-19 currently exists. Drug repurposing of available antiviral agents may provide a respite; moreover, a cocktail of antiviral agents may be helpful in treating this disease. Here, we have highlighted a few available antimicrobial agents that could be very effective in treating COVID-19; indeed, a number of trials are underway to detect and confirm the efficacy of these agents.

## Introduction

In December 2019, the city of Wuhan, China, saw the outbreak of an unusual disease manifesting as severe pneumonia and respiratory distress. This disease epidemic was later shown to be caused by severe acute respiratory syndrome coronavirus 2 (SARS-CoV-2) and has now engulfed the world. The disease has spread across borders, leading to a global pandemic, and is currently showing no significant plateauing. SARS-CoV-2, formerly known as novel coronavirus (2019-nCOV), is a positive single-stranded RNA virus belonging to the family coronaviridae (1). Currently, there is not much strong evidence from randomized clinical trials to show improved outcomes or a decrease in terms of mortality with regards to the various treatment options available or prophylactic treatment. With little known about the virus and treatments available, we here highlight some of the leading therapeutic options and compare and contrast these in an attempt to determine which may be the most promising. We try to highlight up-to-date published clinical data and the treatment strategies for this novel pandemic so far.

Given the rapid spread so far and the new treatment modalities under consideration, the main focus lay on the repurposing of existing drugs, with several trials underway in attempts to find the most revolutionizing one.

## Treatment Modalities Compared and Contrasted

The treatment options available for COVID-19 and their mechanisms of action are briefly outlined in Table 1.

**Table 1:** Different drugs available for COVID-19.

**DRUGS**	**Mechanism of action**	**Adult dose/administration drug**	**Contraindications**	**Toxicities**	**References**
1-Choloroquine (CQ)/Hydroxy-Choloroquine (HCQ)	Interfere with viral entry & exit through cell and disruption of viral protein synthesis	Oral, HCQ 400 mg BID × 2 doses, then 400 mg q day × 4 days (five doses)	- Known hypersensitivity to CQ/HCQ and 4-aminoquinoline - Presence of retinal or visual field defects	- CQ/HCQ has a narrow therapeutic index - Can cause QT interval prolongation, torsade de pointes, arrhythmia - Bone marrow suppression - Seizure - Retinopathu - Myopathy	(2–13)
2- Azithromycin	Inhibits bacterial protein synthesis and also has some antiviral effect	Oral, 500 mg × 1, then 25 0 mg × 4 days (5 days total)	- Myasthenia Gravis - Hypokalemia - Hypomagnesemia - Tosade de pointes	- QT interval prolongation	(14–16)
3- Remdesivir	An adenosine analog; causes premature termination of the nascent viral RNA chains by incorporating into the viral genome	IV, 200 mg × 1, then 100 mg daily × 9 days (10 doses)		- Elevated level of transaminases - Kidney injury	(17–24)
4- Lopinavir/Ritonavir	An inhibitor of HIV type 1 protease (HIV-1); halts HIV-1 maturation and thus infectivity; the same for SARS-CoV-2	Oral, 400/100 mg BID × 10 days		**Common:** - GIT intolerance - Nausea/vomiting **Major:** - Pancreatitis - Hepatotoxicity - Cardiac conduction abnormalities	(25–36)
5- Favipiravir	Inhibits RNA-dependent RNA polymerase (RdRp) of RNA viruses which leads to chain termination	Oral, dosage varies Dosage adjustment requires in renal and liver diseases		- Neutropenia - Diarrhea - Hyperuricemia - Elevated level of transaminases	(37–43)
6- Ribavirin	A nucleoside analog of guanosine, inhibits RNA polymerase and acts as a chain terminator; is incorporated into the genome and causes mutations resulting in defective viral progeny - called “error catastrophe”	Oral, 400 mg TID (>50 ml/min), 400 mg BID (50–30 ml/min), 200 mg daily (<30 ml/min) × 10 days	- Pregnant women - Men whose female partners are pregnant - Patient with hemoglobinopathies	- Hemolytic Anemia - Tretogenic	(44–63)
7- Ivermectin	Anti-parasitic drug; has been shown to halt the replication of SARS-CoV-2 *in vitro*, as indicated by several-fold reduction of viral RNA			- Skin rash - Joint or muscle pain	(64–67)
8-Immunoglobulin	Antibodies obtained from recovered patients of COVID-19 can neutralize the virus when injected into new patients			- Flushing - Headache - Malaise - Fever - Renal impairment - Thrombosis - Arrhythmia	(68–76)
9- Corticosteroids	Corticosteroids play an anti -inflammatory role because of their various effects on various cytokines (1L-1, 1L-6, 1L-8, 1L-12, TNFα) and reduce pathological damage		- Patients with underlying infections - Diabetes - Hypertension	Short-term use does not cause any significant side effects, but long-term use can result in: - hypertension - diabetes - Osteoprosis - Weight gain	(77–81)
10. Interferon	Proteins secreted by cells of the immune system; boost the immune system			- Flu-like symptoms such as headache, fatigue, and weakness - Chills - Fever	(82–91)
11. Tocilizumab	Recombinant human IL-6 monoclonal antibody; binds to IL-6 receptors	IV, 400 mg (flat dose) × 1	- Patients with known hypersenstivity to tocilizumab - Caution in patients with neutropenia (<500 cells/micro L) or thrombocytopenia (<50,000 cells/micro L)	- Increase in upper respiratory tract infections like tuberculosis - Nasopharyngitis - Headache - Hypertension - Hematologic effects - Hepatotoxicity -GIT perforation - Hypersensitivity reactions	(92–102)

### Chloroquine (CQ)/Hydroxychloroquine (HCQ)

CQ and HCQ have been used for the treatment of malaria for 70 years. CQ and HCQ act on multiple pathways of virus entry into and exit from cells and cause disruption of the essential viral protein synthesis (2). The *in-vitro* activities of CQ and HCQ have been shown to have an inhibitory effect on SARS-CoV-2 mRNA production, with HCQ showing greater efficacy than CQ (3, 4). However, *in vitro* activity cannot be interpreted as clinical activity against COVID-19; *in vitro* activity of CQ/HCQ against many other viruses, such as Ebola virus (5), Chikungunya virus (6), influenza virus (7), HIV (8), and dengue virus (9), has been reported previously, but their clinical efficacy did not reach that seen *in vitro*.

In a non-randomized trial in France on 36 patients with COVID-19, HCQ was administered alone or in combination with azithromycin. After 6 days of treatment, 100% of patients treated with the hydroxychloroquine and azithromycin combination had no detectable viral load in nasopharyngeal swabs compared to 57.1% of patients treated with hydroxychloroquine only and 12.5% of the control group (*p* < 0.001) (10). In another report from China in 100 patients with COVID-19, those treated with HCQ showed better clinical outcomes than control patients (11).

Triggered somewhat by the media and an intense pressure to prescribe a medication to COVID-19 patients and also due to the general perception of CQ/HCQ efficacy, clinicians may turn to off-label use of CQ/HCQ. Off-label use of CQ/HCQ is occurring globally, including in some hospitals in the USA but should be approached cautiously, as CQ and HCQ have a narrow therapeutic index and can cause QT interval prolongation, torsade de pointes, arrhythmia (12), bone marrow suppression, seizure, retinopathy, and myopathy.

Given the lack of evidence, we strongly call on public health organizations to collaborate effectively with local governments to support unified randomized controlled trials (RCTs) to test the potential therapeutic effects of CQ/HCQ against COVID-19. If the ethical use, safety, and advanced clinical efficacy of CQ/HCQ can be established by RCTs, as proposed by the WHO, it would be a significant advancement in the treatment of COVID-19 patients. Global multicenter RCTs would be the most effective approach for collecting accurate data about the safety and clinical efficacy of CQ/HCQ for the treatment of COVID-19, and this strategy would allow robust data to be available in the near future (13).

### Azithromycin

Azithromycin is a bacteriostatic belonging to the macrolides class that inhibits bacterial protein synthesis and thus interferes with bacterial growth. It is also known to have antiviral effects in addition to its antibacterial properties. It has been used to treat respiratory viral infection due to this former property (14).

In a small non-randomized study conducted by Gautret et al., azithromycin in combination with HCQ has demonstrated substantial antiviral activity against SARS-CoV-2 (10). Literature on azithromycin alone as a treatment option for COVID-19 is scarce, and it is not clear whether macrolides can be used alone or should be in combination with HCQ. Masashi et al. believe that macrolides alone, or in combination with other drugs, are effective against SARS-CoV-2 (15).

Several clinical trials are being conducted to check the efficacy of HCQ-azithromycin for SARS-CoV-2. An interventional clinical trial is underway to determine the efficacy and safety of HCQ-azithromycin (16).

### Remdesivir

Remdesivir is an adenosine analog that interferes with the synthesis of new viral RNA by chain termination. Although it was developed to be used against Ebola virus and Marburg virus infections (17), it showed antiviral activity against many other RNA viruses such as Lassa virus, respiratory syncytial virus, and coronaviruses such as SARS-CoV and MERS-CoV. Due to its antiviral activity against SARS-CoV and MERS-CoV, it has also been tested against SARS-CoV-2.

Remdesivir achieved satisfactory results in the Ces1c (–/–) mouse SARS model. It significantly reduced lung virus titers and improved pulmonary function when administered one day after disease onset (*p* < 0.0001). Virus titers were discernibly reduced, but with high mortality of mice, when administered 2 days after disease onset. This study concluded that when lung injury reaches a peak, simply reducing the virus titers can no longer suppress the strong immune responses in mice, but remdesivir can significantly improve the symptoms and mortality (*p* = 0.0037) in mice when administered at early stages (18).

A case has been reported in which a patient with a SARS-CoV-2 infection confirmed by RT-PCR (performed on a nasopharyngeal swab) showed drastic improvement in one day with remdesivir (19). On account of the broad-spectrum anti-CoV activity of remdesivir, a randomized, double-blinded clinical trial was planned and is still ongoing (20). This study includes 308 participants, randomized to either remdesivir or placebo. Another phase 3 randomized, double-blinded, placebo-controlled study is underway focusing on the safety and efficacy of remdesivir in 452 hospitalized adults with severe respiratory symptoms from SARS-CoV-2 (21).

In an *in vitro* study, remdesivir inhibited the growth of bat-CoVs and human CoV (22). Another study revealed that remdesivir and chloroquine are very effective against SARS-CoV-2 *in vitro* (23).

Preliminary results from a recent randomized, placebo-controlled, double-blind phase 3 clinical trial in hospitalized patients with COVID-19 revealed that compared to placebo, remdesivir was associated with shorter time to recovery (11 vs. 15 days) (24)

### Lopinavir/Ritonavir

Lopinavir is an inhibitor of HIV type 1 protease (HIV-1), halting the maturation of HIV-1 and thus its infectivity (25). Ritonavir, which is also a protease inhibitor, is administered in combination with lopinavir to enhance its bioavailability by inhibiting its metabolic inactivation (25). This combination is considered to be a highly effective antiretroviral agent, and some studies even advocate the use of monotherapy as a therapeutic option in certain HIV-infected patients (26). Along with other drugs (chloroquine, chlorpromazine, and loperamide), lopinavir was found to inhibit the *in vitro* replication of MERS-CoV and SARS-CoV (27). In patients with SARS associated with SARS-CoV infection, the combination of lopinavir/ritonavir and ribavirin resulted in a lower rate of acute respiratory distress syndrome (ARDS) or death at day 21 when compared to the historical control group treated with ribavirin only (2.4 vs. 28.8%, *p* < 0.001) (28). The lopinavir/ritonavir and ribavirin combination also allowed a reduction in steroid dosages and resulted in a decreased incidence of nosocomial infection (28). Lopinavir/ritonavir also reduced mortality in marmosets with MERS-like disease. The mortality rate at 36 h post-inoculation was 0–33% with lopinavir/ritonavir treatment vs. 67% in untreated or mycophenolate-treated animals (29). A case was reported in which a patient with MERS-CoV pneumonia improved and showed viral clearance after 6 days of triple antiviral therapy with lopinavir/ritonavir, ribavirin, and pegylated interferon (IFN)-alpha 2a (30). In another case, a patient with MERS-CoV pneumonia who later developed renal failure was started on triple antiviral therapy (lopinavir/ritonavir, ribavirin, and pegylated interferon) and showed resolution of viremia 2 days after treatment initiation, though virus shedding continued, highlighting the importance of starting ribavirin treatment early (31).

Given the effectiveness of lopinavir/ritonavir against MERS-CoV and SARS-CoV, it was thus tested for the treatment of SARS-CoV-2. Lopinavir, but not ritonavir, inhibits the *in vitro* replication of SARS-CoV-2 (32). Lopinavir/ritonavir was recommended for the treatment of SARS-CoV-2 pneumonia in China (33). A small report showed that out of four patients (two with mild SARS-CoV-2 pneumonia and two with severe) treated with lopinavir/ritonavir, umifenovir, and Shufeng Jiedu Capsule (a traditional Chinese medicine), three patients showed significant improvement and were discharged, while the other patient (with severe pneumonia) showed signs of improvement (34). In a patient with SARS-CoV-2 mild pneumonia, administration of lopinavir/ritonavir resulted in a decrease in the viral load from the very next day, and viral titers were later undetectable (35). The author highlighted that the decrease in viral titers could be due to the natural course of the disease; therefore, further studies are needed to determine the direct antiviral effect of lopinavir/ritonavir. Another young woman treated with lopinavir/ritonavir for SARS-CoV-2 pneumonia showed improvement after 7 days of therapy (36).

### Favipiravir

Favipiravir inhibits RNA-dependent RNA polymerase (RdRp) of RNA viruses (but not cellular RNA and DNA synthesis) (37) and shows broad-spectrum antiviral activity against RNA viruses (38). Favipiravir (T-705) can induce mutations in the genome of the influenza virus, which reduces the infectivity of the virus *in vitro*. This mechanism of lethal mutagenesis is proposed to be the key antiviral mechanism of favipiravir (39). It was originally developed against the influenza virus (38) and was the first effective drug against Ebola virus infection in an animal model (40). Favipiravir has shown *in vitro* effectiveness against the rabies virus (RABV) but is ineffective *in vivo*, especially after neuroinvasion. Although favipiravir blocked RABV replication at the site of inoculation in mice, it was not effective in the CNS, which means a method for its adequate penetration into the CNS needs to be devised (41). A randomized clinical trial in China comparing the efficacy of favipiravir and umifenovir for moderate symptoms showed that favipiravir is superior to umifenovir, having a higher recovery rate (71.4 vs. 55.9% for favipiravir and umifenovir, respectively; *p* = 0.0199). The time to cough relief and fever reduction by favipiravir was also shorter than that by umifenovir (*p* < 0.0001) (42). Further clinical trials of favipiravir in adult patients with SARS-CoV-2 pneumonia have been approved in China (43), and similar trials are being conducted at Harvard University and also in Japan.

### Ribavirin

Ribavirin is a guanosine analog that acts as a chain terminator by inhibiting RNA polymerase (44). Alternative potential mechanisms could include its incorporation into the HCV genome, causing mutations and resulting in the production of defective viral progeny in a process called “error catastrophe” (45), or the inhibition of inosine monophosphate dehydrogenase (46). It is being used in combination with interferon in patients with chronic hepatitis C (47) and showed good results in patients with respiratory syncytial virus, especially immunocompromised patients (48).

The use of ribavirin in addition to corticosteroids in patients with SARS-CoV pneumonia resulted in resolution of fever and lung opacities within 2 weeks (49). In another study in Canada, ribavirin was administered to patients with clinical improvement, but no clear benefit was found. However, this study did highlight the side effects of ribavirin, as 49% of the patients showed a decrease in hemoglobin levels of 2 g/dL, and 76% showed signs of hemolysis, diagnosed by a 1.5 times increase in bilirubin or decreased haptoglobin level (50). In a series of 31 patients with SARS, 1 patient recovered with antibiotics only, 17 showed a rapid response to combination therapy (ribavirin and methylprednisolone), while the remaining required step-up or pulsed methylprednisolone therapy. This highlights the importance of ribavirin therapy in SARS (51). Ribavirin, when used in combination with interferon β, inhibits SARS-associated coronavirus replication *in vitro*; in another study, it showed antiviral activity against SARS coronavirus when used synergistically with interferon 1α and interferon β (52, 53). In a further study, it was able to lower the viral load in five out of eight patients (54). Ribavirin and interferon α2a given to MERS-CoV patients resulted in 14/20 (70%) survival as compared to 7/24 (29%) survival with no treatment at day 14 (*p* = 0.04) but 6/20 (30%) survival in the treatment group vs. 4/24 (17%) survival with no treatment at day 28 (*p* = 0.54). There was no significant difference between the later groups (55). It did not show any advantages in SARS-CoV patients (56, 57). Ribavirin used with interferon α in MERS-CoV resulted in improvement in 4 days in one patient and 6 days in another (58). No treatment advantage was seen with ribavirin in MERS-CoV after meta-analysis (59, 60). Ribavirin showed *in vitro* antiviral effects against SARS-CoV-2 (24). Ribavirin can be used for COVID-19 (61), and it has also been recommended to use for COVID-19 via intravenous infusion (62), as it binds tightly with SARS-CoV-2 RdRp and stops polymerase function (63). However, we need a randomized control trial to elucidate the antiviral potential of ribavirin. Moreover, it should be used in combination with either interferon or lopinavir/ritonavir to enhance its antiviral activity against SARS-CoV-2.

### Ivermectin

This FDA-approved anti-parasitic drug has been shown to halt the replication of SARS-CoV-2 *in vitro*, as indicated by a several-fold reduction in viral RNA ivermectin-treated samples (64). However, further evaluation is needed to determine its efficacy in combating COVID-19 in humans. Ivermectin also shows broad-spectrum antiviral activity. It inhibits yellow fever virus replication, specifically targeting NS3 helicase activity (65). Ivermectin also inhibits HIV-1 (66) and Dengue virus (66, 67) replication by inhibiting importin alpha/beta, which facilitates the transport of proteins between the cytoplasm and nucleus, as these viruses use these proteins for their replication.

### Immunoglobulin

IgG antibodies have two functional parts: Fab fragments, which help in antigen recognition, and the Fc fragment, which helps in the activation of the immune system (68). Intravenous immunoglobulin (IVIG) is effectively used for autoimmune diseases and chronic inflammatory diseases such as lupus, multiple sclerosis, Kawasaki disease, and dermatomyositis (69, 70). It has been used for the treatment of various bacterial, viral, and fungal infections in humans and in many experimental models (71, 72).

Likewise, SARS-Cov-2 infections could be treated using polyclonal antibodies from recovered COVID-19 patients (73). It would be preferable to extract the immunoglobulin from patients in the same city or the same area, as lifestyle, diet, and the environment are implicated in the development of specific antibodies against the virus. Immune IgG collected in China may be different from that collected in Europe or the USA (74).

In an uncontrolled case series, five critically ill COVID-19 patients on ventilators and receiving methylprednisolone and antiviral agents were transfused with convalescent plasma containing SARS-CoV-2 specific antibody (IgG) at a binding titer >1:1,000 that had been obtained from five recovered COVID-19 patients. Convalescent plasma was transfused between days 10–22 after admission. Out of five patients, three were weaned from the ventilator after 2 weeks, and four out of five recovered from ARDS after 12 days of transfusion of convalescent plasma. Three patients were discharged after 51-, 53-, and 55-days stays at hospital, and the remaining two were in stable condition after 37 days of convalescent plasma transfusion (75). There were a few limitations to the above study. Firstly, this was a small case series with no control patients, and secondly, these patients had already been given antiviral agents and steroids.

This method of passive antibody therapy can provide an effective treatment against the rapidly rising pandemic of COVID-19 (76). Though serum antibodies have been in use as a treatment for a relatively long time, further clinical trials with control groups are needed to support the idea of using serum antibodies as a treatment option for COVID-19.

### Corticosteroids

Corticosteroids are a class of steroid hormones that play a key physiological role in inflammation and the immune system. The use of corticosteroids for COVID-19 has been controversial since the outbreak of this disease (77). In the past, corticosteroids have been widely used for treatment during SARS-CoV outbreaks because of their effects on various cytokines [IL-1, IL-6, IL-8, IL-12, and tumor necrosis factor-α (TNF-α)] (78, 79). Studies in humans have shown that corticosteroids are effective in reducing pathological damage, but the main concern is their adverse effects, such as acute respiratory syndrome (79).

A study was conducted on the treatment of porcine respiratory coronavirus with dexamethasone and showed that one or two doses at earlier stages are effective in reducing pro-inflammatory responses but prolonged use may play a role in enhancing viral replication (80). Another Chinese study was conducted in which SARS-CoV patients were divided into four groups; this showed that early and high doses of steroids with quinolone had an effective response (56).

A randomized clinical trial will be conducted to determine the effectiveness of systemic glucocorticoids in patients with severe novel coronavirus pneumonia (6). The use of corticosteroids for COVID-19 is controversial because of the risks of acute respiratory syndrome and further enhancement of viral replication (81).

### Interferon

Interferons are naturally occurring proteins produced and secreted by cells of the immune system, e.g., white blood cells, epithelial cells, and fibroblasts. There are three major classes of interferon (alpha, beta, and gamma). Each class has a different and diverse action. Interferons boost the immune system against invading antigens, such as viruses and bacteria, and affect not only the stimulated cell but also neighboring cells (82).

Literature reviews highlight that interferons have been in use for many years against emerging viruses when no other treatment options have been available (83). Interferons have also been used for SARS-CoV and MERS-CoV in the past and have shown promising results both *in vitro* and *in vivo* in decreasing viral replication (83–86). Most of the time, interferons were used in combination with ribavirin or lopinavir/ritonavir, but the potential benefits did not meet expectations, most probably because of their administration at later, post-infectious, stages (59).

From previous studies, we can assume that interferons may be an effective treatment option against SARS-CoV-2 (87). SARS-CoV and MERS-CoV are able to disrupt interferon signaling pathways by interfering with proteins involved in interferon expression, such as Orf6 and Orf3b (88). The excessive *in vitro* sensitivity of SARS-CoV-2 to interferons is potentially because SARS-CoV-2 might have lost these anti-interferon actions due to their truncated Orf6 and Orf3b proteins (89). This suggests that interferons may be a better potential treatment option for SARS-CoV-2 than for SARS-CoV. As interferon treatment is more effective at earlier stages, they can be used prophylactically against SARS-CoV-2, and this is further supported by the *in vitro* efficacy of interferon pretreatment against the virus (89). Shen et al. stated that interferon-2α can effectively reduce the infection rate of SARS-CoV-2, which further supports the above hypothesis (90).

The recommended guidelines for the treatment of SARS-CoV-2 in China include administering 5M units of interferon α via an inhaler in combination with oral ribavirin twice a day (62). The advantage of inhalation therapy is that it acts directly on the respiratory tract; however, the pharmacokinetics and pharmacodynamics of this route are not precisely known (87). A clinical trial is underway to determine the effectiveness of interferon α with ribavirin and lopinavir/ritonavir in COVID-19 patients (91).

Due to the greater sensitivity of SARS-CoV-2 to interferon α in comparison to its family members (SARS-CoV and MERS-CoV), it can be used as an effective treatment option for COVID-19 patients. However, it will be necessary to wait for the results from current clinical trials to understand the exact efficacy of interferon (87).

### Tocilizumab

Tocilizumab is a recombinant anti-human interleukin (IL)-6 receptor monoclonal antibody. It binds to both membrane-bound and soluble IL-6 receptors (IL-6R) and prevents further inflammatory cascades (92). It has been seen that critical SARS-CoV-2 patients have a surge of inflammatory cytokines, called a cytokine storm, as was previously seen with SARS and MERS. These inflammatory markers (IL-1B, IL-1RA, IL-7, IL-8, IL-9, IL-10, fibroblast growth factor, granulocyte-macrophage colony stimulating factor, IFNγ, granulocyte-colony stimulating factor, interferon-γ-inducible protein, monocyte-derived growth factor, TNFα, and vascular endothelial growth factor) were high in COVID-19 patients, leading to systemic inflammation and multi-organ failure (93, 94). IL-6 and IL-2 receptor (IL-2R) can be used to predict the severity of COVID-19-related pneumonia, as significant differences between the levels of IL-6 and IL-2R were seen between the three clinically differentiated groups (*p* < 0.05). The study showed that the more severe the disease, the higher the levels of IL-6 and IL-2R (95). Several other reports have shown elevated levels of IL-6 in COVID-19 critical patients (96). IL-6 is a key substance in cytokine release syndrome, so blocking IL-6R with tocilizumab can save patients with severe COVID-19 (97). In a small clinical trial in China, 21 patients with severe or critical COVID-19 were treated with tocilizumab. Within a few days of treatment, fever resolved in all patients, 15/20 (75%) needed less oxygen (one needed no oxygen), and CT scans showed resolution of pulmonary lesions in 19/21 (90.5%) patients. Lymphocytes in 10/19 (52.6%) patients and C-reactive protein (CRP) in 16/19 (84.2%) patients also returned to normal (98). In another retrospective study, tocilizumab was administered to 15 patients, and a significant improvement was seen in CRP levels, which dropped from 126.9 (10.7–257.9) to 11.2 (0.02–113.7) mg/L (*p* < 0.01) (99). Many individual cases have been reported in which the use of tocilizumab resulted in a significant improvement in patients. A 60-year-old patient with a previous history of multiple myeloma presented with chest tightness, and his chest CT showed multiple ground-glass opacities. He was admitted and given moxifloxacin for 3 days. Later, he was given umifenovir (arbidol), as the diagnosis of COVID-19 was confirmed by real time RT-PCR performed on a nasopharyngeal swab.

Two weeks later, he was transferred to another hospital as his chest tightness had worsened, and his oxygen saturation had become low. A CT scan showed bilateral, multiple ground-glass opacities. He was given methylprednisolone on days 2–6 of admission to improve his chest tightness and dyspnea. The patient still had bilateral, multiple ground-glass opacities on a chest CT performed on day 8. His laboratory results showed a high level of serum IL-6, and he was administered 8 mg/kg of IV tocilizumab. His IL-6 level started decreasing, chest tightness improved, and his CT on day 19 showed a decrease in ground-glass opacities (100).

Another 42-year-old patient with a history of renal cell carcinoma presented with fever and received ceftriaxone. After 6 days, he developed cough and fever, and his real-time PCR results for SARS-CoV-2 were positive. A further CT scan showed bilateral ground-glass opacities, and he was started on lopinavir/ritonavir (for 5 days) on day 7. On day 8, he developed dyspnea with decreasing oxygen saturation and was put on oxygen supplementation. He was given two doses of tocilizumab, 8 h apart, and his condition started improving. On day 12, partial regression of pulmonary infiltrates and ground-glass opacities was seen on chest CT. His CRP (a marker of cytokine storm) decreased from 225 mg/L to 33 mg/L in 4 days (101).

A 45-year-old male patient with a history of sickle cell disease presented with vaso-occlusive crises, no pulmonary findings, no dyspnea, no cough, no fever, and oxygen saturation of 98%. On day 1, the patient developed fever, and his oxygen saturation dropped to 91% with auscultatory crepitations. He was given amoxicillin-clavulanic acid and hydroxychloroquine, while a specimen was sent for RT-PCR testing for SARS-CoV-2. On day 3, his saturation dropped to 80% and his chest CT showed abnormal findings consistent with SARS-CoV-2-related pneumonia and acute chest syndrome. After RT-PCR results indicated SARS-CoV-2, a single dose of tocilizumab was injected and the patient improved and was discharged after blood transfusion (his hemoglobin was low) (102).

These cases highlight that tocilizumab can be used to successfully treat COVID-19 patients with respiratory failure by limiting cytokine-related pulmonary damage.

## Vaccines

Vaccination can be the only definitive and preventive treatment option for COVID-19. A number of vaccine clinical trials are being conducted. A clinical trial by the University of Oxford is currently in phase 2/3 (103), and another phase 2 trial by the Institute of Biotechnology, Academy of Military Medical Sciences, PLA of China, is in progress. (104).

## Conclusion

After reviewing a number of studies and case reports, we conclude that remdesivir and hydroxychloroquine/chloroquine with or without azithromycin are promising treatment options for patients with mild and moderate COVID-19. However, tocilizumab and immunoglobulin therapy seem to be effective in treating severe disease. There is a need for randomized control trials involving the entire globe to determine the efficacy and potency of these available potential treatment options.

## Author Contributions

All authors listed have made a substantial, direct and intellectual contribution to the work, and approved it for publication.

## Conflict of Interest

The authors declare that the research was conducted in the absence of any commercial or financial relationships that could be construed as a potential conflict of interest.
